# Effect of motivational interviewing in hypertensive patients (MIdNIgHT): study protocol for a randomized controlled trial

**DOI:** 10.1186/s13063-019-3486-1

**Published:** 2019-07-09

**Authors:** Luana Claudia Jacoby Silveira, Graziella Badin Aliti, Elisabeth Meyer Da Silva, Ravi Pereira Pimentel, Miguel Gus, Eneida Rejane Rabelo-Silva

**Affiliations:** 1 Graduate Program in Health Sciences: Cardiology and Cardiovascular Sciences, Universidade Federal do Rio Grande do Sul, Rua Ramiro Barcelos 2400, Porto Alegre, RS 90035-003 Brazil; 2 Nursing School, Universidade Federal do Rio Grande do Sul, Rua São Manoel 963, Porto Alegre, RS 90620-110 Brazil; 3 Cardiology Division, Hospital de Clínicas de Porto Alegre, Rua Ramiro Barcelos 2350, Sala 2060, Porto Alegre, RS 90035-903 Brazil; 4 Graduate Program, Instituto de Cardiologia - Fundação Universitária de Cardiologia, Rua Princesa Isabel, 395, Porto Alegre, RS 90620-001 Brazil

**Keywords:** Nursing, Motivational interviewing, Hypertension, Randomized clinical trial, Lifestyle

## Abstract

**Background:**

Only one-third of hypertensive patients achieve and maintain blood-pressure control. This is attributed to low treatment adherence and has a negative impact on clinical outcomes. Adherence is multidimensional and involves aspects both related to patient characteristics and to the chronic nature of the disease. In this context, motivational interviewing has been proposed as an approach to foster patients’ motivations to change their behavior for the benefit of their own health, thus providing more lasting behavioral changes.

**Design and methods:**

Single-center, parallel, randomized controlled trial with outcome-assessor blinding. This study will select adult patients (*n* = 120) diagnosed with hypertension who receive regular follow-up in a specialized outpatient clinic. Patients will be randomly allocated across two groups: the intervention group will have appointments focused on motivational interviewing, while the control group will have traditional appointments. Patients will be monitored face-to-face, once monthly for six months. The primary outcomes will be a reduction of at least 8 mmHg in systolic blood pressure and changes in mean blood pressure measured by 24-h ambulatory blood pressure monitoring. Secondary outcomes include improvement of adherence to a low-sodium diet, adherence to self-care behaviors, regular use of antihypertensive medications, increase or maintenance of physical activity, weight reduction, evaluation of changes in daytime sleepiness, and cessation of smoking.

**Discussion:**

This study shows an intervention strategy that will be tested and, if effective, warrant replication in monitoring of other chronic diseases.

**Trial registration:**

ClinicalTrials.gov, NCT02892929. Registered on 24 August 2016.

**Electronic supplementary material:**

The online version of this article (10.1186/s13063-019-3486-1) contains supplementary material, which is available to authorized users.

## Background

Hypertension is a highly prevalent cardiovascular risk factor, as well as the most common condition seen in primary care. Uncontrolled hypertension is the main risk factor for cardiovascular disease, which, in turn, is the leading cause of death in the world [1, 2]. Studies have demonstrated that increased risk starts at blood pressure (BP) values as low as 115/75 mmHg, doubling with each 20 mmHg rise in systolic blood pressure (SBP) or 10 mmHg rise in diastolic blood pressure (DBP) [3].

Poor BP control is associated with low treatment adherence [4–6]. However, because it is a multidimensional phenomenon, adherence is not determined only by issues inherent to patients themselves; disease-related factors, health beliefs, lifestyle, and cultural habits also play important roles [6, 7]. Approximately two-thirds of patients treated for hypertension fail to achieve and maintain adequate BP control [8, 9]. Studies suggest that adherence to self-care behaviors and lifestyle modifications can reduce BP levels [4, 8].

Several interventions to promote lifestyle modifications and self-care behaviors have been proposed. Motivational interviewing (MI), developed by Miller and Rollnick, is a person-centered, directive counseling method designed to stimulate and strengthen personal motivation for change by exploring and resolving ambivalence [10–12]. It is a guiding style meant to enhance intrinsic motivation to change, develop autonomy, and promote behaviors in the interest of health [11, 13]. The practice of MI is based on the following guiding principles: listen with empathy; avoid argumentation and confrontation; roll with resistance; and sustain effectiveness and optimism [13].

In 2008, Ogedebbe et al. tested, in a randomized controlled trial (RCT), the effect of MI counseling versus usual care on medication adherence and BP in 190 African Americans with hypertension. The intervention group received standard care plus four sessions of MI at three-month intervals for a period of one year. The primary outcome was adherence to prescribed antihypertensive medication; the secondary outcomes were changes in office BP, self-efficacy, and intrinsic motivation at baseline and 12 months. The authors found no significant reductions in SBP between groups and no improvement in medication adherence in the intervention group [14]. More recently, in 2012, Ogedegbe et al. tested positive affection induction and self-affirmation strategies as a potential means of improving adherence to drug therapy by African American patients with hypertension. Their results suggested that this intervention was more effective in improving adherence to antihypertensive drug therapy compared with patient education alone [15]. In 2016, Boutin-Foster et al. assessed the effect on BP control of a combination of positive affection and self-affirmation strategies (including MI) compared with conventional educational measures in hypertensive African American patients over a one-year period, with bi-monthly telephone follow-up. This study had several limitations, e.g. at baseline patients in the intervention and control groups both received an educational workbook with the etiology of hypertension, treatment options, and lifestyle changes that one could make to improve BP control and to support goalsetting, and the same research assistants led appointments in the intervention and control groups. The results were not positive for a reduction in BP [16].

In the context of other chronic co-morbidities, Creber et al. compared MI versus usual care in patients with heart failure. The intervention consisted of one home-based motivational interview and three or four follow-up calls. The authors reported improvement of self-care in the MI group [17].

The few existing studies carried out using this intervention showed limited effects on BP lowering with the MI approach. However, some results obtained to date were difficult to generalize because they were tested in a specific population (hypertensive African Americans); other studies used a combination of MI and other strategies; in others, interventions were not face-to-face. Furthermore, none used 24-h ambulatory blood pressure monitoring (ABPM) to measure BP; ABPM findings are now known to be associated more strongly with cardiovascular outcomes than office measurements [18, 19]. We designed this RCT to compare the effectiveness of MI versus usual care in reducing SBP and DBP, measured by ABPM, in patients with hypertension. The intervention will consist of an exclusive MI approach applied monthly in face-to-face appointments over a six-month follow-up period.

## Methods/Design

### Study design

The “effect of MotIvational iNterviewing In HyperTensive patients” (MIdNIgHT) study is a randomized, single-center, parallel clinical trial with a follow-up period of six months. Patients with hypertension will be enrolled from Hospital de Clínicas de Porto Alegre (HCPA), a center in southern Brazil, and invited by telephone to participate in the trial. After a baseline evaluation, participants will be randomly allocated into two groups: (1) an MI intervention group (IG); and (2) a control group (CG) to receive usual care. MI sessions and standard clinical assessments (usual care) will be conducted face-to-face once monthly for six months. Anthropometric measurements, questionnaires and scales (Self-Care of Hypertension Inventory [SC-HI], Medication Assessment Questionnaire [MAQ], Dietary Sodium Restriction Questionnaire [DSRQ], International Physical Activity Questionnaire [IPAQ], Epworth Sleepiness Scale [ESS]), and ABPM will be performed at baseline and at the end of follow-up.

**Fig. 1:**
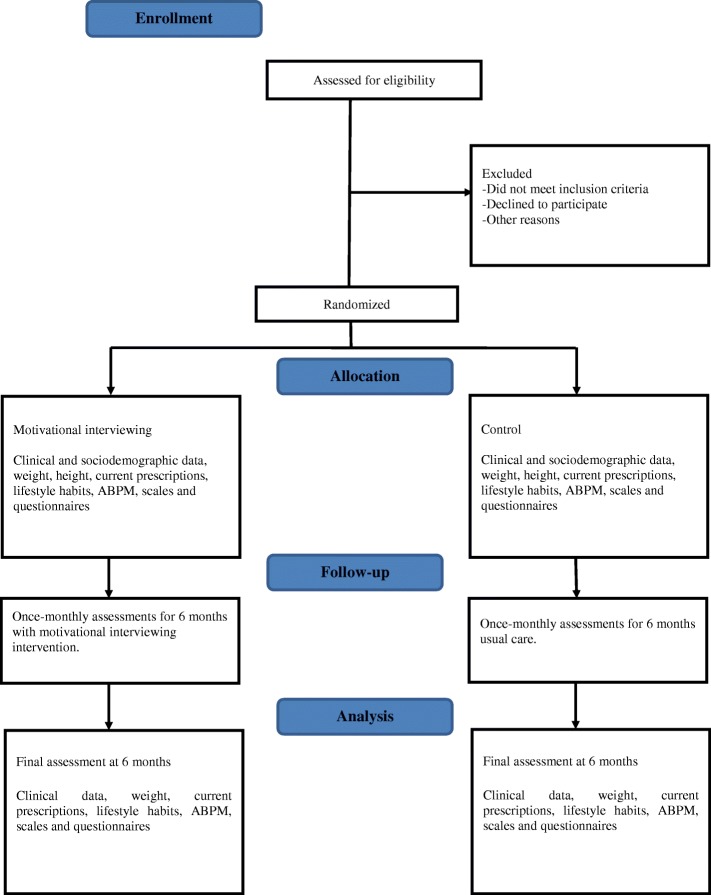
*Flow diagram* of the study

**Fig. 2:**
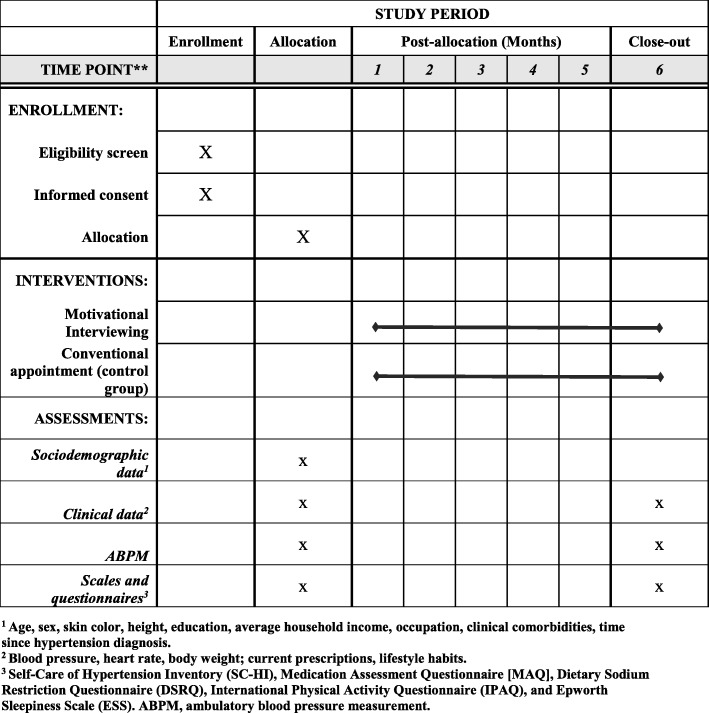
Schedule of enrollment, interventions, and assessments

A flow diagram of the study design is presented in Fig. 1. The study timeline and schedule of enrollment, interventions, and assessments (Standard Protocol Items: Recommendations for Interventional Trials [SPIRIT] figure) and the SPIRIT Checklist are provided as Fig. 2 and Additional file 1, respectively.

### Inclusion and exclusion criteria

The study will include female and male adults (aged 18–80 years) who have been receiving treatment and follow-up at the HCPA outpatient hypertension clinic for > 6 months. Patients must be on at least two antihypertensive medicaments at the time of recruitment and must not have been seen by a nutritionist or followed nutritional guidance in the last six months.

The exclusion criteria will be: pregnancy or lactation; gastrointestinal tract disease; inflammatory disease; chemotherapy; diagnosed diabetes; incapacity to engage in an interview due to intellectual disability or dementia, defined by application of a cognitive deficit assessment questionnaire [20]; and/or incapacity to participate in the intervention program without the need for third-party involvement.

### Ethical considerations

All patients will read and sign an informed consent form before inclusion (Additional file 2). The study protocol was approved by the HCPA Research Ethics Committee with opinion number 160225, registered in the ClinicalTrials.gov database with accession number NCT02892929, and will be conducted in accordance with the principles of the Declaration of Helsinki and the Ethical Principles for Medical Research Involving Human Subjects of the National Health Council of Brazil [21].

### Outpatient hypertension clinic and staff approach

The HCPA outpatient hypertension clinic is a multidisciplinary clinic attended by approximately 150 hypertensive patients per month. The clinic provides regular monitoring to optimize and encourage adherence to drug therapy and management of hypertension-related co-morbidities. Appointments are led by specialized cardiologists, pharmacists, psychologists, researchers, and graduate students.

### Lead researcher training

The lead researcher is a specialist cardiovascular nurse with 10 years of experience in the care of patients with heart disease and will be responsible for application of the MI technique during appointments in the intervention group. She has participated in an intensive MI workshop which followed the recommendations of the Motivational Interviewing Network of Trainers [22] in 2013 and, more recently, in 2015, 2016, and 2018, participated in workshops on advanced MI techniques. During the study, monthly supervision will be provided by another MI specialist. Schwalbe et al. [23] showed that 3–4 post-workshop supervision sessions over a period of six months were enough to maintain the effects of training on MI skills. Training and the supervision will be the responsibility of a PhD researcher in Psychiatry with longstanding expertise in MI and personal training conducted in the United States with Miller and Rollnick.

### Sample size

The sample size was calculated with WinPepi 11.20 software. Delta values of BP levels for the study population as reported by Correa et al. (4.4 mmHg for 24-h SBP and 2.7 mmHg for 24-h DBP) were used [24]. The sample size was estimated at 100 patients, 50 in the IG, and 50 in the CG, considering a difference in SBP of 8 mmHg between groups, a standard deviation of 14 mmHg, 80% power, and alpha = 0.05. To account for 20% attrition, the sample will consist of 120 patients, 60 in each group.

### Study protocol

Patients will be identified by the HCPA hypertension clinic appointment schedule and invited to participate in the study. The first appointment will serve to obtain informed consent and confirm eligibility. Then, the patient will be randomized to either IG or CG. Participants of both groups will attend a total of six monthly appointments, scheduled by prearrangement, at the HCPA Clinical Research Center. At the first and last protocol visits, the clinical and sociodemographic questionnaire and the other study instruments will be applied, anthropometric measurements will be obtained, and the 24-h ABPM device will be placed. Each appointment will have an average duration of 30 min. Patients will be reminded of consultations the day before via phone calls; missed appointments will be rescheduled. Appointments should take place within a one-week interval of the 30-day time point since the preceding visit. The primary outcome of the study will be a reduction of at least 8 mmHg in SBP and changes in DBP and mean BP, all assessed by 24-h ABPM. The secondary outcomes will include improvement of adherence to a low-sodium diet, adherence to self-care behaviors, regular use of antihypertensive medications, improvement or maintenance of levels of physical activity, changes in daytime sleepiness, weight loss, and cessation of smoking. These outcomes will be assessed by appropriate questionnaires (described in detail below); weight loss will be measured directly during visits and cessation of smoking will be self-reported. Analysis will follow the intention-to-treat principle. All collected data will be stored in Microsoft Excel spreadsheets and tabulated by the double data entry method. The results of the trial will be communicated to the participants in a meeting organized at the end of the study.

### Randomization, allocation, and confidentiality

The first study visit will consist of confirmation of eligibility, completion of the informed consent form, and collection of sociodemographic and clinical data. After this initial visit, patients will be allocated to the CG or IG by simple random sampling, using opaque numbered envelopes, each containing a randomization code. The list of computer-generated randomization codes, generated online in blocks of six at <http://www.randomization.com>, will remain in the possession of an independent investigator not otherwise involved in the study.

### Study variables, blinding, and methods of assessment

The research team will be responsible for the screening and selection of eligible participants. Three team members will obtain written informed consent before study enrollment. Researchers will not be blinded to participant intervention. However, the researcher responsible for application of the MI technique and the one responsible for conducting conventional appointments will be blinded to the outcomes of the study. The participants will be blinded to group allocation and primary outcome. The investigators who are not outcome-blinded will be trained to obtained standardized anthropometric and BP measurements as well as to administer the study questionnaires.

### Clinical and sociodemographic variables

A structured questionnaire will be administered to all participants by a trained provider to collect clinical and sociodemographic data (age, sex, skin color, education, average household income, occupation, clinical co-morbidities, time since hypertension diagnosis, time followed at the outpatient clinic, current prescriptions, current BP [two isolated office measurements], heart rate, weight, height, and life habits).

### Anthropometric measurements

The anthropometric measurements of interest will be body weight, height, waist circumference (measured at the midpoint between the lowest rib and the superior edge of the iliac crest), and body mass index. All will be measured on the first and last protocol visits.

### Twenty-four-hour ambulatory blood pressure monitoring

ABPM will be conducted with periodically calibrated devices (DINAMAPA-2000, Cardio Systems Coml. Indl. Ltda.), with cuff size selected in accordance with the patient’s arm circumference. The protocol includes BP measurements every 15 min during the daytime (06:00 to 22:00) and every 30 min during the nighttime (22:00 to 06:00). ABPM will be considered satisfactory if at least 16 valid readings during the daytime and eight valid readings during the nighttime are obtained [18]. Participants will be evaluated by ABPM at the baseline and at the end of the follow-up (six months) in order to register BP during both sleep and wakefulness.

### Questionnaires and scales

The participants will answer the following questionnaires: Dietary Sodium Restriction Questionnaire (DSRQ) [25] for the assessment of sodium-restriction adherence; Self-Care of Hypertension Inventory – Brazilian Version (SC-HI) [26, 27] to measure self-care in hypertension patients; Medication Assessment Questionnaire [MAQ] [28] to evaluate adherence to drug therapy; International Physical Activity Questionnaire (IPAQ) [29] to quantify the level of physical activity; and the Epworth Sleepiness Scale (ESS) [30] to measure daytime sleepiness. All questionnaires will be administered at baseline and at the end of follow-up.

### Allocation

#### Intervention group

The scales and questionnaires defined in the study protocol will be administered at the first and last visits, as will ABPM. Since the first appointment, MI techniques will be used with the aim of working out the ambivalence involved in seeking treatment and modifying unhealthy behaviors, such as physical inactivity, unhealthy diet, tobacco use, alcoholic beverage intake, weight gain, stress, and non-adherence to pharmacological therapy. At all six face-to-face appointments, the MI approach will be applied with a focus on behavior change. Care will be provided in the MI style, which is characterized by being collaborative and non-coercive—for example, using open-ended questions, avoiding argumentation, and practicing empathy and reflective listening to encourage patients to talk about why, when, and how they might change the target behavior. The nurse will disclose the discrepancies that arise when the patient understand the differences between his current situation and his hopes for the future. In addition, the nurse will support self-efficacy to help the patient identify his skills and apply them in the context of hypertension. Finally, the nurse will also summarize what the patient wants to reach behaviorally. The guide which will be used during MI, based on the original methodology proposed by Miller and Rollnick, is shown in Table 1 [10–13, 22].

**Table 1:** Guide for motivational interview appointment

Key points	
Acts by activating the patient’s own motivation for change and adherence to treatment	
Approach is patient-centered	
Collaborative spirit, evocative, and with respect for patient autonomy	
Allows exploring and solving ambivalence	
Offer critical resources that provide the space for a natural change	
Techniques	Skills
- Open questions	- Empathy
- Establish schedule with the patient	- Avoid arguments
- Reflective listening	- Talk about behavior change
- Request permission to inform	- Draw attention to discrepancies
- Stimulate the plan of action	- Respect autonomy
- Summarize the conversation	- Empower the patient
- Use evaluation scales	- Joint decision-making process
- Resist the reflex of repairing unhealthy behaviors	

The motivational interviewing counseling for each ambivalent behavior included the following steps:

(1) Assess the patient’s motivation and confidence on subjective scales of 1–10, to observe motivation to modify ambivalent behaviors and to evoke conversations about change

(2) Detect facilitators for changing these behaviors

(3) Elicit the “pros” and “cons” of any concerns

(4) Provide a menu of options to address any barriers or concerns about improving behaviors. The patient is asked about solutions for any barriers that present; if the patient does not present any barriers, the investigator encourages the patient to maintain their current behavior

(5) Assess the patient’s values and goals. This helps to create ambivalence between current behaviors and goals/values. Patients are asked to sort a list of values in terms of personal importance and to select around five that are most important. They are then asked to briefly discuss why the values/goals selected are important to them and then to explore what connection, if any, they see between their current health behavior and their ability to achieve these goals or live out these values

(6) Establish an action plan and make an overall summary of the appointment. The appointment, when appropriate, ends with an action plan to change the patient’s behavior, and the investigator summarizes what was talked and agreed upon and incorporates the patient’s suggestions and values

These strategies were reinforced during every monthly appointment

#### Control group

Patients allocated to the CG are expected to attend the same number of appointments as those in the IG: a total of six conventional (prescriptive) patients encounters, once monthly, with a nutritionist specializing in cardiology with no knowledge of the MI technique. During each CG appointment, patients will receive general recommendations for hypertension, such as increasing their intake of fruits and vegetables, reducing salt intake, avoiding processed and high-sodium foods, losing weight, and reducing their consumption of alcoholic beverages. Scales and questionnaires will be applied, as will ABPM at the first and last appointments, per protocol.

### Outcomes of interest

The primary outcome of the study will be a reduction of at least 8 mmHg in SBP and changes in DBP and mean BP. These outcomes will be measured by 24-h ABPM at the first and last protocol visits.

Secondary outcomes will include improvement of adherence to a low-sodium diet, adherence to self-care behaviors, regular use of antihypertensive medications, increase or maintenance of physical activity, weight reduction, evaluation of changes in daytime sleepiness, and cessation of smoking. These outcomes will be measured by the aforementioned questionnaires and scales at the first and last protocol visits.

### Statistical analysis

Continuous variables will be expressed as mean ± standard deviation or median and interquartile range. Categorical variables will be expressed as percentage or proportion. The chi-square test will be used for associations between sociodemographic and clinical variables with SC-HI scores. Student’s t-test or the Mann–Whitney *U* test, depending on data distribution, will be used for between-group comparison of quantitative variables. SBP and DBP measurements obtained during the study, as well as SC-HI scores, will be analyzed by generalized estimating equations with Bonferroni correction. BP differences between groups during treatment will be analyzed by repeated-measures analysis of variance (ANOVA). Analysis of covariance (ANCOVA) will be conducted to adjust possible differences regarding baseline measurements, as well as some risk factors and confounding factors when necessary. The 5% level of significance will be adopted and data will be analyzed in IBM SPSS Statistics for Windows, Version 20.0 (IBM Corp., Armonk, NY, USA).

## Discussion

This protocol will test MI as a strategy for reducing BP levels in patients with hypertension through lifestyle modifications. This approach differs from the traditional, prescriptive model used to monitor hypertensive patients at outpatient clinics, in which the patient has little decision-making power, appointments are provider-centered, and management plans are usually presented to patients with little or no discussion of whether they are viable in the patient’s routine. In the IG, once-monthly face-to-face MI visits will be held. These appointments will focus on the patient, who will hear himself talk about the importance of lifestyle modifications and define with the provider the most effective ways to achieve adequate living habits and thus facilitate management of hypertension. If effective, this technique warrants replication in the management of other chronic diseases.

### Potential biases

This study has some limitations. The questionnaires and scales used to measure secondary outcomes are self-reported and thus subject to a range of biases, such as regarding credibility. The open-label design also introduces some potential for bias and the sample size limits the external validity.

### Trial status

The study is currently in the patient recruitment stage. To date, 50 patients have been included and seven patients have already completed all protocol steps.

## Additional files

Additional file 1: SPIRIT 2013 Checklist: Recommended items to address in a clinical trial protocol and related documents. (DOC 123 kb)

Additional file 2: Consent form. (DOCX 13 kb)

## Data Availability

Not applicable.

## Abbreviations

ABPM: Ambulatory blood pressure monitoring
BP: Blood pressure
CG: Control group
CRC: Clinical Research Center
DBP: Diastolic blood pressure
GEE: Generalized estimating equations
HCPA: Hospital de Clínicas de Porto Alegre
IG: Intervention group
MI: Motivational interviewing
RCT: Randomized clinical trial
SBP: Systolic blood pressure
