# Supplementary material for: Effect of motivational interviewing in hypertensive patients (MIdNIgHT): study protocol for a randomized controlled trial
Source: Trials. 2019 Jul 9;20:414. doi: 10.1186/s13063-019-3486-1 (PMC6617897; doi:10.1186/s13063-019-3486-1)
Supplement: Supplementary file 2 — Consent form. (DOCX 13 kb) [file 13063_2019_3486_MOESM2_ESM.docx]

**Additional file 2- Consent Form**

**Consent Form**

**Project: Effect of Motivational Interview as a Strategy for Reducing Blood Pressure in Hypertensive Patients.**

Hospital de Clínicas de Porto Alegre Research Ethics Committee

Rua Ramiro Barcelos, 2350 – 2nd Floor – phone: 3359-7604.

You are being invited to participate in scientific research that aims to evaluate the effect of a new approach to clinic visits, the Motivational Interview, on reducing blood pressure in patients with hypertension.

By agreeing to participate in the survey, you will be asked some questions. You will return to the hospital five times, once a month. At each consultation, your blood pressure and weight will be checked and you will completed health assessment scales. During the first and last visits, you will receive a blood pressure measurement device for 24-hour ABPM.

The 24-hour ABPM is performed by placing a blood pressure monitor (sphygmomanometer) on your arm, where it will stay for 24 hours, inflating and deflating to check your blood pressure every 15 minutes.

You may have all the information you want and are free to not participate in the survey or withdraw your consent at any time, without detriment to your care. By participating in the study, you will not receive any financial compensation, but we will ensure that all expenses necessary for the study, except for transportation to and from the Center, will not be your responsibility. Your name will not be disclosed to other people, because you will be identified by a number or a letter. The information we obtain will be used for research purposes only. Please be advised that any possible risk or discomfort involved in this research are related to the time you will have to respond to the survey questionnaires during the first and last consultation, you will benefit from a more thorough blood pressure assessment through the tests that will be performed during the study, and you will receive the results of your evaluation.

I, __________________________________________, have read and / or heard the above clarification, and have understood the reason for the study and the procedure to which I shall be subjected. The explanation I received clarifies the risks and benefits of the study. I understand that I am free to stop participating at any time without having to justify my decision and that this will not affect my treatment. I know that my name will not be disclosed, that I will not bear any expenses, and will not receive any money for participating in the study. I agree to participate in this study.

The investigator responsible for this research project is Prof. Graziella Badin Aliti, and the student who will lead the project is Nurse Luana Claudia Jacoby Silveira. This document was reviewed and approved by the Research Ethics Committee of this institution.

If you have any questions, you can contact the researchers at (51) 8159-9582 or 3359-8499/3359-7604.

Porto Alegre, ____­­­­­­­­­____________.
